# Supplementary material for: Molecular and microbiological evidence of bacterial contamination of intraocular lenses commonly used in canine cataract surgery
Source: PLoS One. 2022 Nov 21;17(11):e0277753. doi: 10.1371/journal.pone.0277753 (PMC9678303; doi:10.1371/journal.pone.0277753)
Supplement: S1 Table — Average percentages and standard deviation of the most abundant bacterial groups for each manufacturer based on 16S rRNA sequencing. (DOCX) [file pone.0277753.s001.docx]

| *AnVision* | | | |
| --- | --- | --- | --- |
| **IOL** | | **Packaging fluid** | |
| Taxon  **Phylum**  -Family  --*Genus* | Avg.  abundance  (% ± SD) | Taxon  **Phylum**  -Family  --*Genus* | Avg. abundance (% ± SD) |
| **Firmicutes**  -Stapylococcaceae  --*Staphylococcus spp.* | 31.7 ± 18.6 | **Firmicutes**  -Staphylococcaceae  --*Staphylococcus spp.* | 42.7 ± 20.5 |
| *Dioptrix* | | | |
| **IOL** | | **Packaging fluid** | |
| Taxon  **Phylum**  -Family  --*Genus* | Avg.  abundance  (% ± SD) | Taxon  **Phylum**  -Family  --*Genus* | Avg. abundance (% ± SD) |
| **Firmicutes**  -Staphylococcaceae  --*Staphylococcus spp.* | 12.9 ± 16.2 | **Firmicutes**  -Staphylococcaceae  --*Staphylococcus spp.*  -Bacillaceae  --*Bacillus spp.* | 9.4 ± 8.1  8.5 ± 8.6 |
