# Supplementary material for: Molecular and microbiological evidence of bacterial contamination of intraocular lenses commonly used in canine cataract surgery
Source: PLoS One. 2022 Nov 21;17(11):e0277753. doi: 10.1371/journal.pone.0277753 (PMC9678303; doi:10.1371/journal.pone.0277753)
Supplement: Attachment — Submitted filename: Response to Reviewers.docx [file pone.0277753.s002.docx]

The authors wish to sincerely thank the reviewer for their time and editorial suggestions to our manuscript. We sincerely appreciate the fact that the reviewer appears to be as enthusiastic about this data as we are. Our responses to each comment can be found below in **blue**. We have made all suggested changes in the manuscript where appropriate.

Review Comments to the Author

Please use the space provided to explain your answers to the questions above. You may also include additional comments for the author, including concerns about dual publication, research ethics, or publication ethics. (Please upload your review as an attachment if it exceeds 20,000 characters)

Reviewer #1: This study investigated the presence of bacteria and/or bacterial DNA contamination in presumably sterile and commercially available canine IOLs and their packaging fluid. I appreciate your efforts in this important and emerging area of research. The manuscript is well-written, concise, and informative. The number of products sampled for each manufacturer is relatively small, but as a descriptive pilot study this is adequate. Further clarification regarding negative controls and data filtering methods are necessary before publication.

Thank you kindly for your time and effort in reviewing our manuscript. Please see our comments below regarding the use of data filtering methods in our updated manuscript.

Materials & Methods:
For consistency, please have the order of experiments in the Materials & Methods match the Results. For example, if conventional bacterial culture is mentioned first in the Materials & Methods, please list it first in the Results.
Thank you for bringing this to our attention, and we have modified the Results section to improve consistency of the manuscript.

Although the authors have used negative controls to assess the presence of environmental contaminants, which can significantly influence samples from sites with low microbial biomass, it is not clear if contaminant filtering analysis was conducted to discount the possibility of reagent/environmental contaminants impacting the results. Information regarding subtractive filtering should be provided in the manuscript. More information is provided below.
We concur, and thank you for pointing this out. As part of the revision process, we have utilized the decontam package to censor contaminant taxa from our dataset. The retained amplicon sequence variants are considered to be truly present in the intraocular lenses and packaging fluid. We have revised the data within the manuscript, figures, and tables to reflect the updated dataset.

Results:
Table S1 – “Uncultured” bacterium is likely inappropriate terminology for microbiome data. Consider renaming to unidentified or unlisted bacterium.
Thank you for bringing this to our attention, and we have modified Table S1 accordingly.

Discussion:
243-249: As mentioned above, subtractive filtering to remove contaminants is important for low biomass samples, especially since the negative controls closely resemble the IOL and fluid packaging samples in this study. Several methods have been developed to identify and remove contaminating DNA, and this should be utilized and described in the manuscript. For example, Decontam is an open-source R package that identifies contaminants in microbiome data to censor contaminant taxa from the dataset allowing researchers to generate more accurate profiles of microbial communities. Following the use of any subtractive filtering technique, the read count of each sample should be assessed to determine whether the minimum cutoff based on the rarefaction curve has been met. Samples with very low read counts should be excluded from further analysis.
Thank you for bringing this to our attention. Please see our comments above regarding the use of decontam to remove contaminant DNA in our dataset. Our data, manuscript, figures, and tables have been updated accordingly. Additionally, the reference (listed below) has been added to revised manuscript regarding the script used to filter out contaminant sequences.

Davis NM, Proctor DM, Holmes SP, Relman DA, Callahan BJ. Simple statistical identification and removal of contaminant sequences in marker-gene and metagenomics data. Microbiome. 2018;6(1):226.
[https://doi.org/10.1186/s40168-018-0605-2](https://nam02.safelinks.protection.outlook.com/?url=https%3A%2F%2Fdoi.org%2F10.1186%2Fs40168-018-0605-2&data=04%7C01%7Cdowlerk%40mail.missouri.edu%7Cc928cfeafdb84ae63a7b08da0e8d8a26%7Ce3fefdbef7e9401ba51a355e01b05a89%7C0%7C0%7C637838298307511790%7CUnknown%7CTWFpbGZsb3d8eyJWIjoiMC4wLjAwMDAiLCJQIjoiV2luMzIiLCJBTiI6Ik1haWwiLCJXVCI6Mn0%3D%7C3000&sdata=EU6weO6bOcgzLa0Yt6ynseNmdbO8MA18F0UvKRWhSkc%3D&reserved=0)
